# Synthesis, characterization, and preclinical validation of a PET radiopharmaceutical for interrogating Aβ (β-amyloid) plaques in Alzheimer’s disease

**DOI:** 10.1186/s13550-015-0112-4

**Published:** 2015-05-24

**Authors:** Guruswami SM Sundaram, Dhruva Dhavale, Julie L Prior, Jothilingam Sivapackiam, Richard Laforest, Paul Kotzbauer, Vijay Sharma

**Affiliations:** ICCE Institute, Molecular Imaging Center, Box 8225, 510 S. Kingshighway Blvd., St. Louis, MO 63110 USA; Mallinckrodt Institute of Radiology, Washington University School of Medicine, Box 8225, 510 S. Kingshighway Blvd., St. Louis, MO 63110 USA; Department of Biomedical Engineering, Box 8225, 510 S. Kingshighway Blvd., St. Louis, MO 63110 USA; Department of Neurology, Washington University School of Medicine, Box 8225, 510 S. Kingshighway Blvd., St. Louis, MO 63110 USA

**Keywords:** PET imaging, Radiopharmaceuticals, β-amyloid (Aβ), Alzheimer’s disease

## Abstract

**Background:**

PET radiopharmaceuticals capable of imaging β-amyloid (Aβ) plaque burden in the brain could offer highly valuable diagnostic tools for clinical studies of Alzheimer’s disease. To further supplement existing armamentarium of FDA-approved agents as well as those under development, and to correlate multiphoton-imaging data reported earlier, herein, we describe preclinical validation of a PET tracer.

**Methods:**

A novel PET radiopharmaceutical (^18^F-**7B**) was synthesized and characterized. To assess its affinity for Aβ, binding assays with Aβ_1-42_ fibrils, Alzheimer’s disease (AD) homogenates, and autoradiography studies and their IHC correlations were performed. For assessing its overall pharmacokinetic profiles in general and its ability to cross the blood-brain barrier (BBB) in particular, biodistribution studies in normal mice were performed. Finally, for evaluating potential for ^18^F-**7B** to serve as a targeted Aβ probe, the microPET/CT imaging was performed in age-matched amyloid precursor protein/presenilin-1 (APP/PS1) mice and wild-type (WT) counterparts.

**Results:**

The radiotracer ^18^F-**7B** shows saturable binding to autopsy-confirmed AD homogenates (*K*_d_ = 17.7 nM) and Aβ_1-42_ fibrils (*K*_d_ = 61 nM). Preliminary autoradiography studies show binding of ^18^F-**7B** to cortical Aβ plaques in autopsy-confirmed AD tissue sections, inhibition of that binding by unlabeled counterpart **7A-**indicating specificity, and a good correlation of tracer binding with Aβ immunostaining. The agent indicates high initial penetration into brains (7.23 ± 0.47%ID/g; 5 min) of normal mice, thus indicating a 5-min/120-min brain uptake clearance ratio of 4.7, a benchmark value (>4) consistent with the ability of agents to traverse the BBB to enable PET brain imaging. Additionally, ^18^F-**7B** demonstrates the presence of parental species in human serum. Preliminary microPET/CT imaging demonstrates significantly higher retention of ^18^F-**7B** in brains of transgenic mice compared with their WT counterparts, consistent with expected binding of the radiotracer to Aβ plaques, present in APP/PS1 mice, compared with their age-matched WT counterparts lacking those Aβ aggregates.

**Conclusions:**

These data offer a platform scaffold conducive to further optimization for developing new PET tracers to study Aβ pathophysiology *in vitro* and *in vivo*.

## Background

Alzheimer’s disease (AD) is the most frequent form of dementia which affects 24 million people worldwide and also lacks effective therapeutic interventions [1]. Without successful treatment or prevention, the number of affected individuals can be expected to grow exponentially to 13 to 16 million in the United States and to >100 million globally by 2050. Overall healthcare cost in the United States alone in 2050 has been projected to be more than $1 trillion [2]. The failure of clinical drug trials to reverse clinical symptoms indicates that, for a given treatment to be effective, it most likely needs to be prescribed at a preclinical stage before the symptomatic expression of the disease. Therefore, there is an urgent need to identify and validate biomarkers that are present at preclinical stages. Importantly, several biomarkers identified for diagnosis, staging, and assessment of therapeutic effects are (but not limited to): amyloid deposition; changes in CSF levels of tau; hyperphosphorylated tau (p-tau), or Aβ_1-42_; and reduced metabolism monitored via fluorodeoxyglucose (FDG) PET imaging [3–6]. While amyloid deposition and variations in CSF levels of tau and Aβ represent pathophysiological markers thus relevant for disease diagnosis, the reduced metabolism (FDGPET) or atrophy (MRI) demonstrate topographic markers indicating a progression of the disease. Furthermore, literature precedents of the last decade indicate that AD pathological changes (Aβ deposition and NFT formation) occur years prior to onset of symptoms [7]. For diagnosis of AD, several PET radiopharmaceuticals such as [^11^C]2-(4′-methylaminophenyl)-6-hydroxybenzothiazole, ([^11^C]PIB) [8], 2-(1-{6-[(2[^18^F]fluoroethyl)(methyl)-amino]-2-naphthyl}Autoethyli-dene)-malononitrile ([^18^F]FDDNP) [9], [^11^C]4-N-methylamino-4′-hydroxystilbene (SB-13) [10], and (E)-4-(2-(6-(2-(2-(2-^18^F-fluoroethoxy)ethoxy)-ethoxy)pyridin-3-yl)vinyl)-N-methyl benzenamine ([^18^F]Avid45) [11], and 2-(2-fluoro-6-(methylamino)pyridine-3-yl)benzofuran-6-ol(^18^F-AZD4694) [12, 13] have been investigated in humans. In addition, [^125^I/^131^I]TZDM and [^125^I]IMPY have also been investigated for SPECT applications [9]. While [^11^C]PIB has been most intensely studied, [^18^F]Avid45 (Amyvid) [14], ^18^F-Flutemetamol (Vizamyl) [15, 16], and Florbetaben (Neuraceq™) [17–19] have been recently approved by FDA for Aβ imaging. Importantly, both Amyvid [20] and [^11^C]PIB show promising results in humans and excellent correlation with FDG [8]. Recent investigations using PIB and Amyvid [21] binding to AD homogenates also indicate multiple binding sites on Aβ [22], thus mandating development of new tracers to study Aβ pathophysiology. To further supplement the existing armamentarium of FDA-approved Aβ-imaging agents; recently, we have shown that a heterocyclic fluorescent molecule **7A** is able to traverse the blood-brain barrier (BBB) to label Aβ plaques in brains of APP^+/−^/PS1^+/−^ mice and also labels diffuse plaques in autopsy-confirmed AD human tissues [23, 24]. Herein, we report synthesis and characterization of (E)-5-(2-(6-(2-[^18^F]-fluoroethoxy)-benzo[d]thiazol-2-yl)vinyl)-N,N-dimethylpyridin-2-amine, an F-18-labeled counterpart (^18^F**-7B**), and perform its preclinical validation to evaluate its potential to serve as an Aβ-targeted PET radiopharmaceutical for monitoring plaque burden in AD.

## Methods

All reagents were purchased from Sigma-Aldrich unless otherwise stated. 2-fluoroethyl-4-methylbenzene sulfonate was prepared using literature procedure [25]. ^1^H NMR, proton-decoupled ^13^C NMR, and ^19^F NMR spectra were recorded on a Varian 400-MHz spectrometer; chemical shifts are reported in *δ* (ppm) with reference to either TMS or trichlorofluoromethane (CFCl_3_). Mass spectra were obtained from the University of Missouri Mass Spectrometry facility using nitrobenzyl alcohol (NBA) as matrix and analyzed via HRFab. Purity of the **7A** and **7B** were assessed using an HPLC (Waters system 600 equipped with dual λ-detector 2487 set to 254 and 280 nm) with a C-18 reversed-phase column (Phenomenex Luna® C18; 100 Å; 5 μm; 250 × 10 mm) using an eluent mixture of acetonitrile and water as a gradient system (75% acetonitrile in water over 20 min) at a flow of 3 mL/min.

### Chemistry and radiochemistry

#### (E)-5-(2-(6-methoxybenzo[d]thiazol-2-yl)vinyl)-N,N-dimethylpyridin-2-amine (2)

To the mixture of 6-methoxy-2-methyl benzothiazole (1.0 mmol) and 6-dimethylamino pyridine carbaldehyde (1.0 mmol) in DMSO was added 50% KOH and stirred at room temperature for 12 h. After the completion of the reaction, the reaction mixture was filtered, and the yellow solid obtained was used for the next reaction without purification. Yield 92% (0.28 g); yellow solid; R*f* 0.62 (3:2 hexane-EtOAc); ^1^H NMR (400 MHz, CDCl_3_ ): 3.15 (s, 6H), 3.88 (s, 3H), 6.55 (d, *J* = 8.4 Hz, 1H), 7.04 (d, *J* = 9.2 Hz, 1H), 7.14 (d, *J* = 16.0 Hz, 1H), 7.29 (t, *J* = 14.0 Hz, 2H), 7.71 (d, *J* = 8.4 Hz, 1H), 7.83 (d, *J* = 8.4 Hz, 1H), 8.29 (s, 1H); ^13^C NMR (100 MHz, CDCl_3_): 38.14, 55.79, 104.13, 106.05, 115.31, 118.0, 119.41, 123.02, 134.08, 134.22,148.93, 159.15, 165.33; HRMS (FAB) m/z calc. for C_17_H_18_N_3_OS: [M]^+^ + 1 312.1171; found: 312.1167.

#### (E)-2-(2-(6-(dimethylamino)pyridin-3-yl)vinyl)benzo[d]thiazol-6-ol (3)

The condensed product (0.28 g, 1.0 mmol) was taken in a 50-mL RB, dissolved in dry DCM under argon and subjected to −78°C using dry ice/acetone bath and stirred for 5 min. BBr_3_ (1 M in DCM, 5 mL, 5.0 mmol) was added drop wise maintaining the same temperature. The resulting mixture was slowly brought to room temperature and stirred overnight. The completion of the reaction was monitored by TLC. Once the reaction is completed, the flask is cooled to 0°C before addition of cold satd. sodium bicarbonate solution (5 mL). The reaction mixture is then extracted with ethyl acetate (4 × 25 mL), washed with water (2 × 50 mL), dried over anhydrous sodium sulfate, and the solvent evaporated under reduced pressure to give the red solid which was further purified by flash chromatography using hexane:EtOAc:MeOH (10:9:1) as eluent. Yield 86% (0.27 g); light red solid; R*f* 0.24 (1:1 hexane-EtOAc);^1^H NMR (400 MHz, CDCl_3_): 3.03 (s, 6H), 6.65 (d, *J* = 8.4 Hz, 1H), 6.88 (d, *J* = 7.6 Hz, 1H), 7.24 to 7.32 (m, 2H),7.66 (d, *J* = 8.4 Hz, 2H), 7.90 (d, *J* = 7.6 Hz, 1H), 8.29 (s, 1H), 9.82 (s, 1H); ^13^C NMR (100 MHz, CDCl_3_): 38.04, 106.51, 107.08, 116.11, 118.03, 119.58, 123.17, 134.13, 134.96, 135.60, 147.47, 149.36, 155.90, 159.25, 163.97; HRMS (FAB) m/z calc. for C_16_H_16_N_3_OS: [M]^+^ + 1 298.1014; found: 298.1015.

#### (E)-5-(2-(6-(2-((t-butyldimethylsilyl)oxy)ethoxy)benzo[d]thiazol-2-yl)vinyl)-N,N-dimethylpyridin-2-amine (4)

To the solution of alcohol (0.15 g, 0.4 mmol) and 2-(Bromoethoxy)-tert-butyldimethylsilane (0.096 g, 0.4 mmol) in DMF (5 mL) was added Cs_2_CO_3_ (0.20 g, 0.6 mmol). The resulting mixture was stirred at 140°C for 6 h. Following the completion of the reaction (monitored by TLC), it was quenched with the addition of ice cold water and extracted with ethyl acetate (3 × 25 mL). The organic layer was washed with water (2 × 50 mL) and dried over anhydrous sodium sulfate and the solvent evaporated under reduced pressure to give crude products which were purified by PTLC using hexane:EtOAc (60:40) as eluent; yield 54% (0.12 g); yellow solid; R*f* 0.44 (2:3 hexane-EtOAc); ^1^H NMR (400 MHz, CDCl_3_): 0.12 (s, 6H), 0.92 (s, 9H), 3.15 (s, 6H), 4.02 (bs, 1H), 4.11 (bs,1H), 6.56 (d, *J* = 8.8Hz, 1H), 7.06 (d, *J* = 8.4 Hz, 1H), 7.14(d, *J* = 16.4 Hz, 1H), 7.24 to 7.32 (m, 2H), 7.72 (d, *J* = 8.8 Hz, 2H), 7.82 (d, *J* = 8.8 Hz, 1H), 8.29 (s, 1H); ^13^C NMR (100 MHz, CDCl_3_): 31.04, 42.24, 68.44, 76.22, 110.76, 112.22, 121.21, 124.34, 129.86, 140.02, 142.74, 149.96, 152.44, 162.68, 172.86; HRMS (FAB) m/z calc. for C_24_H_34_N_3_OSSi: [M]^+^ + 1 456.2140; found: 456.2145.

#### (E)-2-((2-(2-(6-(dimethylamino)pyridin-3-yl)vinyl)benzo[d]thiazol-6-yl)oxy)ethanol (5)

To the solution of TBDMS-protected compound (0.05 g, 0.1 mmol) in THF was added TBAF (1 M in THF, 0.5 mL, 0.5 mmol) and stirred at RT for 6 h. Following completion of the reaction (monitored by TLC), the solvent was evaporated under reduced pressure to obtain a crude product which was purified by PTLC using hexane:EtOAc (75:25) as eluent. ^1^H NMR (400 MHz, CD_3_COCD_3_): 3.17 (s, 6H), 3.96 (bs, 2H), 4.20 (bs, 2H), 6.65 (d, *J* = 8.9, Hz, 1H), 6.71 (d, *J* = 8.8 Hz, 1H), 7.10 (t, *J* = 8.2 Hz, 1H), 7.23 (d, *J* = 16.4 Hz, 1H), 7.43 (d, *J* = 16.4 Hz, 1H), 7.57 to 7.93 (m, 2H), 8.36 to 8.42 (m, 1H) ^13^C NMR (100 MHz, CD_3_COCD_3_ ): 37.08, 60.41, 70.33, 104.64, 105.07, 105.82, 115.79, 116.09, 117.76, 119.44, 122.88, 123.21, 133.65, 134.23, 149.04, 150.26, 157.26.

#### (E)-2-((2-(2-(6-(dimethylamino)pyridin-3-yl)vinyl)benzo[d]thiazol-6-yl)oxy)ethyl-4-methylbenzenesulfonate (6)

Pyridine (0.08 g, 1 mmol) and DMAP (0.0012 g, 0.01 mmol) were added to a solution of alcohol (0.08 g, 0.2 mmol) in DCM (10 mL) at 0°C. Thereafter, *p*-toluene-sulfonylchloride (0.076 g, 0.4 mmol) dissolved in DCM (2 mL) was added, and the resulting solution was stirred at room temperature for 7 h and quenched by the addition of water (15 mL). The resulting mixture was extracted with DCM (3 × 5 mL), and organic extracts were combined, dried over Na_2_SO_4_, filtered, and concentrated. Finally, the residue was purified by Prep TLC using the eluent mixture (Hex/EtOAc = 60:40) to obtain the compound as a viscous yellow liquid. Yield 57% (0.06 g); yellow viscous liquid; R*f* 0.41 (1:1 hexane-EtOAc): ^1^H NMR (400 MHz, CDCl_3_): 2.43 (s, 3H), 3.15 (s, 6H), 4.22 (m, 2H), 4.41 (bs, 2H), 6.52 (d, *J* = 8.4 Hz, 1H), 6.54 (d, *J* = 8.4 Hz, 1H), 6.68 (d, *J* = 12.6 Hz, 1H), 6.81 (d, *J* = 12.6 Hz, 1H), 6.91(d, *J* = 8.8 Hz, 1H), 7.08 to 7.17 (m, 1H), 7.30 to 7.34 (m, 1H), 7.71 (d, *J* = 8.8 Hz, 1H), 7.79 (d, *J* = 9.6 Hz, 1H), 7.83 (d, *J* = 9.6 Hz, 1H), 7.95 (d, *J* = 8.8 Hz, 1H), 8.31 (d, *J* = 10.0 Hz, 1H) ^13^C NMR (100 MHz, CDCl_3_ ): 21.63, 38.08, 66.08, 67.99, 104.97, 105.05, 106.05, 115.53, 115.68, 117.82, 120.96, 123.03, 123.46, 127.97, 129.84, 134.10, 134.27, 134.45, 137.89, 144.99, 148.98, 149.76, 156.02. HRMS (FAB) m/z calc. for C_25_H_25_N_3_O_4_S_2_Na: [M]^+^ + Na 518.1184; found: 518.1176.

#### (E)-5-(2-(6-(2-fluoroethoxy)benzo[d]thiazol-2-yl)vinyl)-N,N-dimethylpyridin-2-amine (7A)

Dry tetrabutylammonium fluoride (0.065 g, 0.25 mmol) was dissolved in dry acetonitrile (5 mL) under argon and treated with the acetonitrile solution of tosylated precursor **6** (0.050 g, 0.1 mmol) and refluxed at 110°C. The progress of the reaction was monitored via the TLC. Following completion of the reaction, the solvent was evaporated, and the residue was extracted with EtOAc (2 × 10 mL). Combined organic extracts were dried with Na_2_SO_4_, filtered, and evaporated, and the residue was purified using thin-layer chromatography employing a mobile eluent mixture (Hex:EtOAC = 80:20) to obtain **7A** (0.021 g; 60%; bright yellow solid; R*f* = 0.42; 3:2, EtOAc-hexane).^1^H NMR (400 MHz, CDCl_3_): *δ* 3.14 (s, 6H), 4.20 to 4.33 (m, 2H), 4.79 (dd, *J* = 47.6, 8.0 Hz, 2H), 6.51 to 6.57 (m, 1H), 6.67 to 6.82 (m, 1H), 7.08 (dd, *J* = 9.2, 2.6 Hz, 1H), 7.23 to 7.34 (m, 1H), 7.30 to 7.34 (m, 1H), 7.70 to 7.97 (m, 2H), 8.31 (dd, *J* = 8.8, 2.0 Hz 1H); ^13^C NMR (100 MHz, CDCl_3_ ): *δ* 38.10, 38.13, 67.63, 67.84, 81.01, 82.70, 105.32, 106.05, 115.67, 117.86, 119.34, 123.12, 134.23, 134.33, 148.98, 156.36, 159.17, 165.74; ^19^F NMR (282 MHz, CFCl_3_ ): −224 ppm; HRMS (FAB) m/z calc. for C_18_ H_19_ FN_3_ OS: [M + 1]^+^ 344.1232; found: 343.1230.

### Radiochemical synthesis ^18^F-7B

For radiolabeling, tosylated precursor (**5**, 3 mg) dissolved in dry acetonitrile (400 μL) was transferred into an amber-colored vial containing anhydrous kryptofix 2, 2, 2 (Sigma Chemicals, Perth, WA, AU)/K_2_CO_3_/^18^F-fluoride, obtained using standard procedures. The reaction mixture was heated to 100°C for 20 min in an oil bath, cooled in ice-cold water, and diluted to 5% acetonitrile in water. The crude mixture was loaded on a C-18 sep-Pak cartridge (Waters, Milford, MA, USA), primed with ethanol (5 mL) and water (10 mL). The C-18 sep-Pak was washed with water (5 mL × 6) and 25% acetonitrile (5 mL × 4) and finally eluted with 100% acetonitrile (1 mL).

The crude mixture was purified using high-performance liquid chromatography (dual λ detection set at 254 and 280 nm) equipped with a radiodetector (Bioscans) using a C-18 column (Phenomenex (Torrance, CA, USA); 5-μm 250 × 10 mm) using an eluent gradient acetonitrile 75% to 95% over 20 min (flow rate: 3 mL/min). The fraction of ^18^F-**7B** eluting at 9.5 min (radiochemical purity >95%; radiochemical yield: 30%; specific activity: 1,200 to 1,400 Ci/mmol) was collected, concentrated, resuspended in ethanol/saline, and employed for bioassays.

### Metabolite analysis

Human serum (Sigma-Aldrich, St. Louis, MO, USA) was thawed. Serum (100 μL) was taken, diluted with saline to 10%, and incubated with HPLC-purified fraction of ^18^F-**7B** (100 μCi each) at 37°C for 30 and 60 min. Samples were periodically withdrawn and analyzed on radio-HPLC using eluent mixture described above and radio-TLC using an eluent mixture of ethanol/saline in a ratio of 90:10 (to allow mobility of parental compound and other hydrophobic metabolites off the surface to analyze protein binding) and 10:90 (to analyze hydrophilic metabolites).

### Preparation of human brain homogenates

Well-established literature procedures were used for preparation of AD homogenates [11, 26]. Grey matter was isolated from frozen postmortem frontal cortex tissue by dissection with a scalpel. To prepare insoluble fractions, dissected tissue was sequentially homogenized in four buffers (3 mL/g wet weight of tissue) with glass dounce tissue grinders (Kimble, Vineland, NJ, USA): 1) high salt (HS) buffer: 50 mM Tris-HCl pH 7.5, 750 mM NaCl, 5 mM EDTA; 2) HSbuffer with 1%Triton X-100; 3) HSbuffer with 1%Triton X-100 and 1 M sucrose; and 4) phosphate-buffered saline (PBS). Homogenates were centrifuged at 100,000×*g*; after each homogenization step, the pellet was resuspended, and homogenized in the next buffer in the sequence. For comparison in initial binding studies, unfractionated tissue homogenates were also prepared by homogenization of tissue in only PBS.

### Preparation of Aβ1-42 fibrils

Aβ1-42 fibrils were obtained using literature procedures described earlier [26]. Briefly, synthetic Aβ1-42 peptide (1 mg) (Bachem, Torrance, CA) was initially dissolved in DMSO (50 μL) and diluted with the addition of mQ-H_2_O (925 μL). Finally, 1 M Tris-HCl (25 μL; pH 7.6) was added to the peptide solution to obtain a final peptide concentration (222 μM; 1 mg/mL) [27]. Thereafter, the peptide solution was incubated for 30 h at 37°C with shaking at 1,000 rpm in an Eppendorf Thermomixer. The fibril formation was confirmed by ThioT fluorescence. For determining the concentration of fibrils, the fibril reaction mix was centrifuged at 15,000×*g* for 15 min to separate the fibrils from the monomer. The concentration of Aβ monomer in the supernatant was determined in a BCA protein assay.

### Binding assays

Binding assays were performed using previously described methods [26]. Briefly, a fixed concentration (1 μM/well) of Aβ1-42 fibrils was incubated for 1 h at 37°C with increasing concentrations of ^18^F-**7B** (1.5 to 100 nM) in 30 mM Tris-HCl pH 7.4, 0.1% BSA in a reaction volume of 150 μL. A fixed ratio of hot:cold (^18^F-**7B** and **7A**) was used for all radioligand concentrations. The exact hot:cold (^18^F-**7B**:**7A)** ratio was measured in each experiment by counting an aliquot of a sample (2 μL) of the radioligand preparation in a scintillation counter. Binding of ^18^F-**7B** to human brain homogenates was assessed by incubating samples of insoluble fraction (5-μg insoluble protein/well) from AD subjects, with increasing concentrations of ^18^F-**7B** (1.5 to 100 nM). Nonspecific binding was determined in parallel experiments utilizing **7A (**2.5 μM) for Aβ1-42 fibrils and **7A** (5 μM) for AD tissue as a competitor. Bound and free radioligand were separated by vacuum filtration through glass fiber 96-well filter plates (Millipore Multiscreen FB filter plate), followed by washes using ice-cold assay buffer (3 × 150 μL). Glass fiber filters containing the bound ligand were mixed with Optiphase Supermix scintillation cocktail (150 μL; PerkinElmer, Waltham, MA, USA) and counted immediately. All data were obtained in triplicate and analyzed by curve fitting to a one-site binding model using nonlinear regression employing Graphpad Prism software (version 4.0) to determine *K*_d_ and *B*_max_ values. *B*_max_ values were calculated in pmol/gram wet weight of brain tissue [28]. To calculate binding potential (BP) where BP = *B*_max_/*K*_d_, *B*_max_ was converted from pmol/gram brain tissue to units of nanomolar by assuming 1 g of brain tissue = 1 mL [28].

### Autoradiography and immunohistochemistry

Frozen AD frontal cortex sections (12 μm) were obtained using a TBS Minotome PLUS Cryostat. The tissue sections were thaw-mounted onto Superfrost Plus (Fisherbrand 12-550-15 (Fisherbrand, Leicestershire, UK)) microscope slides, allowed to air dry for 10 to 15 min, and stored at −80°C. For autoradiography, brain sections were brought to room temperature for 5 min and then pre-incubated in assay buffer (30 mM Tris-HCl, pH 7.4 + 0.1% BSA) for 10 min at RT. Sections were incubated with ^18^F-**7B** (300 μL/slide; 10 nM) in the assay buffer for 60 min at room temperature. For determining nonspecific binding, adjacent sections were incubated in the additional presence of 2.5 μM cold ligand **7A**. Sections were then washed at RT for 1 min in 30 mM Tris-HCl pH 7.4, 2 min in 70% ethanol/30 mM Tris-HCl, 1 min in 30% ethanol/30 mM Tris-HCl, and 1 min in 30 mM Tris-HCl (washing protocol using literature precedents [29]. Sections were then dried and exposed to phosphor-imaging screen (BAS-MS 2025) for 30 min. Autoradiography images were obtained on a Fuji Bio-Imaging System FLA-7000 (Tokyo, Japan) and analyzed using MultiGauge software (Fuji, Tokyo, Japan). Following exposure to a phosphor-imaging screen, the sections were then blocked for 60 min at room temperature with 3% milk-0.25% Tween 20-PBS buffer. Immunostaining for Aβ plaques was carried out using monoclonal antibody HJ3.4 (directed against the N-terminus of human Aβ) conjugated to Alexa 568 (generously provided by John Cirrito). The sections were incubated overnight in antibody diluted 1:250 in 0.5% milk-PBS-0.25% Tween 20 at 4°C, washed for 5 min × 3 with PBS-0.25% Tween 20, and air dried [30, 31]. Finally, sections were cover-slipped and scanned with the NanoZoomer 2.0-HT System (Olympus) at 20× resolution using TRITC fluorescence filter, and images were acquired using NDP scan 2.5 software (Olympus).

### Biodistribution studies

All animal procedures were approved by the Washington University Animal Studies Committee. Pharmacokinetics of ^18^F-**7B** in brain and other critical tissues of normal male 12-week-old FVB (wild type (WT); 28 to 36 g) mice were determined as previously described [32]. Briefly, ^18^F-**7B** (740 kBq) was dissolved in 100-μL saline containing 10% ethanol. All animals were anesthetized by isofluorane inhalation and injected with radiotracer ^18^F-**7B** (740 kBq, 100 μL) via bolus injection through a tail vein. Animals were sacrificed by cervical dislocation under anesthesia at 5, 30, 60, and 120 min after injection (*n* = 3 each). Blood samples were obtained by cardiac puncture, organs then harvested rapidly, and all tissue samples analyzed for γ-activity. Data are expressed as the percentage injected dose (%ID) per gram of tissue (tissue kBq (injected kBq)^−1^ (g tissue)^−1^ × 100).

### MicroPET/CT imaging

For imaging, female double-transgenic mouse (BL6/FVB APP/PS1, 22.6 months old, *n* = 3) and a female wild-type mouse (BL6/FVB; 22.6 months old, *n* = 3) were anesthetized with isoflurane (1.5% to 2.5%) in oxygen at flow rate of 1 to 2 L/min via an induction chamber and maintained with a nose cone. Following anesthesia, the mice were secured with their heads in the center of the field of view, fixed in the scanner in a prone head-first position (HFP), and placed in an acrylic-imaging tray. MicroPET imaging was performed using Inveon PET/CT scanner (Siemens Medical Solutions, Malvern, PA, USA) following intravenous tail-vein injection of HPLC-purified [^18^F]**7B** (12.95 MBq; 28 μL; 35% ethanol in saline) employing the catheter system in a slow bolus, followed by flushing with isotonic saline solution. PET dynamic data acquisition was performed over 75 min starting immediately following injection of the tracer. The emission data were normalized and corrected for attenuation, scatter, and decay. Attenuation correction was obtained using the co-registered CT data. The image volume consisted of 256 × 256 × 159 voxels, with a size of 0.39 × 0.39 × 0.8 mm^3^ per voxel for the Inveon scanner. For anatomical visualization, PET images were co-registered with CT images from an Inveon PET/CT scanner. For analysis, brain uptake (Bq/L) was normalized to injected dose and weight of animals. For analysis, PET images were initially reconstructed in 52 dynamic frames and the second time as four frames of 30 min each, with 3D-MAP reconstruction algorithm for incorporating resolution recovery (20 iterations, *β* value of 0.0043). For evaluation of uptake ratios of cortex and cerebellum, the regions of interest (ROI) were drawn in the frontal cortex (target) and cerebellum (reference) regions in both transgenic and WT mice. Additionally, SUV values in time-activity curves were normalized to the average activity measured in those regions between 1 to 2 min post-injection. All image data were processed and analyzed using Inveon Research Workspace 4.1 software (Siemens, Malvern, PA, USA). All PET and CT image datasets were scaled to calibrated kBq/cc.

## Results and discussion

**Scheme 1:**
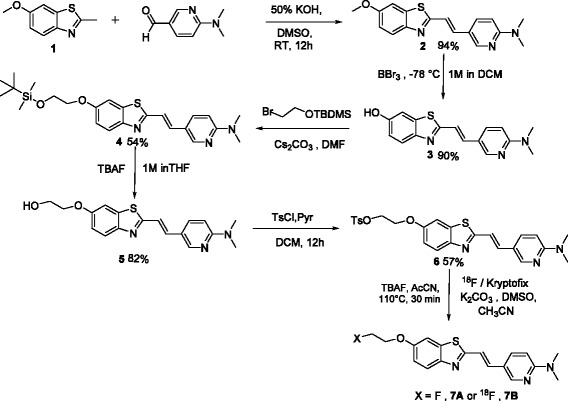
Preparation of the precursor ligand, parental unlabeled ligand (**7A**), and its no-carrier-added counterpart (**[** ^**18**^ **F]-7B**).

**Figure 1:**
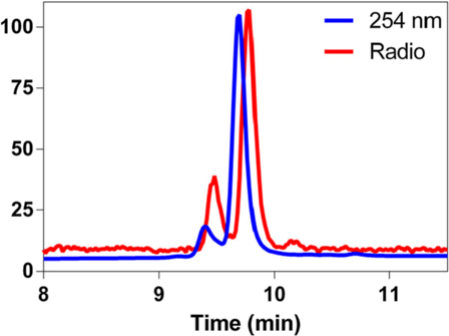
Radio-HPLC data of purified [^18^F]-**7B** spiked with an unlabeled counterpart (**7A**) for characterization of the radiotracer (**7B**).

For synthesis, 6-methoxy-2-methylbenzothiazole **1** was obtained using literature procedures [23, 33] and condensed with 6-(dimethyl-amino)-nicotinaldehyde in an aqueous potassium hydroxide (50%) solution dissolved in DMSO to obtain **2**. Following purification, **2** was demethylated in the presence of BBr_3_ to yield the phenolic derivative **3**. Further, **3** was alkylated with 2-bromoethoxy-t-butyl-dimethylsilane in the presence of cesium carbonate to obtain *t*-butyl-dimethylsiloxy-ethoxy intermediate **4**. Following treatment with TBAF, the intermediate yielded the corresponding ethanol derivative **5**. Upon treatment with p-toluene sulfonylchloride, final precursor ligand **6** was obtained (Scheme 1). While the unlabeled counterpart **7A** was obtained by treatment of precursor ligand **6** with TBAF, the no-carrier-added counterpart PET-tracer ^18^F-**7B** was obtained via standard nucleophilic substitution, using a well-established krytofix/^18^F methodology. While all intermediates **1**, **2**, **3**, **4**, **5**, and **6** were characterized via routine analytical methods, the ^18^F-**7B** was characterized via co-injection with a well-characterized sample of its unlabeled counterpart **7A** (Figure 1). The PET tracer ^18^F-**7B** eluted as a single chemical entity (radiochemical purity >95%), with a retention time of 9.5 min on a semi-preparative HPLC column (Phenomenex Luna® C18; 100 Å) (5 μm, 250 × 10 mm; flow rate, 3 mL/min). The appropriate fraction eluting at 9.5 min was collected, concentrated, dissolved in 10% ethanol in saline, and employed for bioassays (specific activity: *1,200 to 1,400 Ci/mmol*). Although brain-imaging agents post-intravenous injection are known to show extremely facile penetration within the brain, nevertheless, permeation of competing labeled entities could complicate analysis. To assess preliminary stability *in vivo*, ^18^F-**7B** was incubated in human serum at 37°C up to 60 min, and radio-HPLC indicated the presence of primarily the parental compound. Of note, radio-HPLC detects the presence of mobile species thereby allowing immobile species to be retained on the column. Therefore, both radio-HPLC and radio-TLC have been used to allow detection of both polar and nonpolar radiometric metabolites. The radio-TLC demonstrated the presence of the single radiometric peak thus consistent with presence of ^18^F-**7B** under these conditions. Overall, the correlation of radio-HPLC and radio-TLC data indicated the metabolic stability of ^18^F-**7B** in the human serum under these conditions.

**Figure 2:**
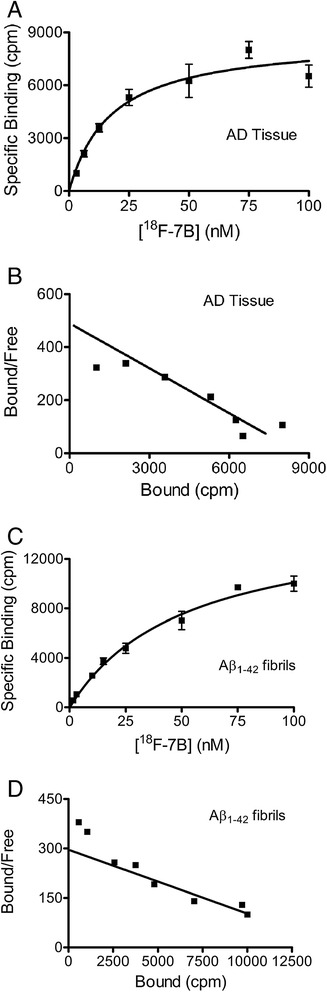
Binding of [^18^F]-**7B** with Aβ_1-42_ fibrils and AD homogenates. AD homogenates (**A**, **B**) and Aβ_1-**42**_ fibrils (**C**, **D**) were incubated with increasing concentrations of [^18^F]-**7B**. Nonspecific binding was determined in parallel experiments utilizing **7A** (5 μM) as a competitor. Representative plots of specific binding versus [^18^F]-**7B** concentration are shown for AD homogenates in (**A**) and Aβ fibrils in (**C**). Data points represent mean ± standard deviations (*n* = 3). The data were analyzed by curve fitting to a one-site binding model using a nonlinear regression. Scatchard plots of the binding data are shown for AD homogenates (**B**) and Aβ_1-42_ fibrils (**D**). Similar results were obtained in more than three independent experiments.

**Figure 3:**
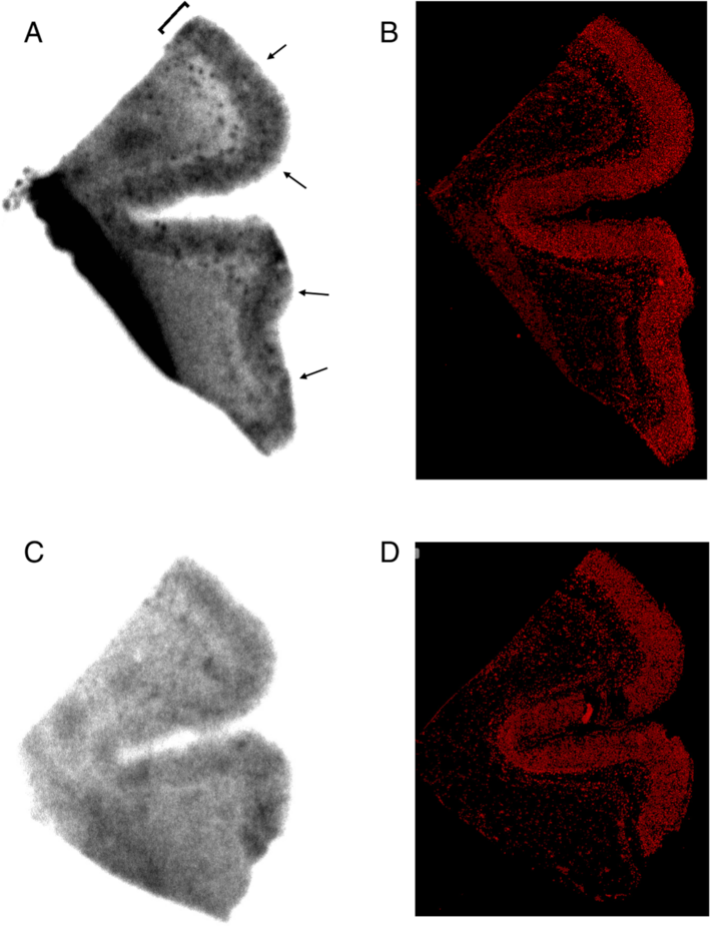
Autoradiography images and fluorescent immunostaining. Autoradiography images of [^18^F]-**7B** binding in an AD frontal cortex section following incubation with [^18^F]-**7B** (10 nM) alone (**A**) or in the presence of **7A** (2.5 μM) (**C**). Fluorescent immunostaining of sections (**A**) and (**C**) with an anti-Aβ antibody is shown in (**B**) and (**D**), respectively. The autoradiography images demonstrate laminar distribution of [^18^F]-**7B** binding in cortex (bracket and arrows), which correlates with the distribution of Aβ plaques shown by fluorescent immunostaining, and binding of [^18^F]-**7B** is inhibited by excess cold ligand (**C**). The higher nonspecific binding in the lower left region (**A**) is due to folding of the tissue section.

For assessing the ability of ^18^F-**7B** to bind Aβ plaques, preliminary binding assays with either preformed Aβ_1-42_ aggregates or AD homogenates were performed in PBS. Nonspecific binding was determined in the presence of **7A** (5 μM) as a competitor. Overall, the binding assay of ^18^F-**7B** with AD homogenates (Figure 2) indicates a saturable specific binding (*K*_d_ = 17.7 nM; *B*_max_ = 1,432 pmol/g wet weight; Figure 2a,b). Similarly, the binding affinity of ^18^F-**7B** with preformed fibrils (*K*_d_ = 61 nM; *B*_max_ = 6 pmol/nmol Aβ; Figure 2c,d) is nearly identical to the *K*_d_ value (59 nM) of **7A** reported earlier [23]. Thus, the radiotracer binding assay technique shows a very close correlation with that of the fluorescence assay indicating identical binding properties of both the labeled and unlabeled counterparts. Furthermore, ^18^F-**7B** shows 3.4-fold higher affinity for AD homogenates compared with that of fibrils, a promising trait of translatable agents. Importantly, binding affinity of ^18^F-**7B** to AD homogenates is 2.6- and 5-fold lower than the FDA-approved radiotracers Flutemetamol [16] and Florbetapir [11], respectively. Furthermore, binding affinity of ^18^F-**7B** is fairly close to that of Florbetaben (*K*_d_: 16 nM), the other FDA-approved radiotracer. However, it can also be argued that any advantage or disadvantage of a given imaging agent attributed only to the binding affinity is also equally dependent upon levels of occurrences of a given biomarker (that could be present either in very low or very high concentrations) in the targeted tissue [34]. One of the guiding principles for designing optimal brain-imaging agents also involves attaining a binding potential (BP) *B*_max_/*K*_d_ ratio ≥10, although a lower value of BP could also enable PET molecular-imaging applications [22, 34, 35]. Given the high concentration of potential Aβ plaques and associated binding sites in AD brain [34], this could also mean that a radioligand with a *K*_d_ value of approximately 18 nM (BP (a combined measure of receptor density and affinity) = 1,432 nM/18 nM = 80) may be sufficient for imaging Aβ in AD. However, it is quite possible that the concentration of potential Aβ binding sites in earliest or presymptomatic AD brain is 1 order of magnitude less than 1,400 nM. Therefore, high-affinity ligands could offer better alternatives specifically when high-affinity binding sites predominate in the cortex and hippocampus of AD homogenates and are absent in the cerebellum [21]. Further analysis of BP of ^18^F-**7B** indicates that its value of 80 (in homogenates) is also 6- and 2-folds lower than that of ^11^C-PIB (*B*_max_: 1,407 nM; *K*_d_: 2.5 nM; BP: 563) [28] and ^18^F-Florbetaben (*B*_max_: 2,431 nM; K_d_: 16 nM; BP: 152), respectively [36]. Combined results of binding affinity data suggest that PET-imaging characteristics of ^**18**^**F-7B** could be slightly inferior to that of Flutemetamol and Florbetapir. Further, to evaluate the ability of ^18^F-**7B** for labeling Aβ in autopsy-confirmed human brain sections, autoradiography and immunohistochemical correlations were also performed. Following incubation of AD frontal cortex sections (12 μm) with ^18^F-**7B** (10 nM) for 60 min, the agent showed labeling of cortical Aβ plaques, and the binding was inhibited upon incubation in the presence of **7A** (2.5 μM) (Figure 3a,c). These data indicate sensitivity and specificity of the ^18^F-**7B**. Additionally, immunohistochemical staining of these sections using anti-Aβ antibody indicated the presence of Aβ plaques in the cortex of these sections (Figure 3b,d), thus demonstrating excellent correlation of immunohistochemical staining data with that of autoradiography data. However, nuclear imaging of a given biomarker *in vivo* is a net function of signal to noise and thus also equally dependent upon pharmacokinetics of a given radiotracer *in vivo*.

Recently [23, 24], we have demonstrated via multiphoton imaging that **7A** could traverse the BBB to label parenchymal plaques in the brain of transgenic mice. However, doses used for fluorescence were several folds higher than required for PET-imaging applications *in vivo*. Therefore, to assess whether or not PET counterpart ^18^F-**7B** administered at tracer concentrations relevant for nuclear imaging also displays similar kinetics *in vivo*, its pharmacokinetic studies in normal mice and microPET-imaging studies in transgenic mice and WT counterparts were performed. To accomplish this objective, biodistribution studies of ^18^F-**7B** were performed in normal FVB mice for preliminary assessment of signal-to-noise ratios and its clearance profiles. Uptake in brain and other critical organs was analyzed in terms of percent injected dose per gram of the tissue (%ID/g) Table 1. For *in vivo* imaging of Aβ plaques, the basic pharmacokinetic model in normal brains involves a high initial penetration of the agent, followed by facile clearance due to lack of a binding target. Preliminary biodistribution studies (Table 1) with HPLC-purified ^18^F-**7B** in normal mice show transient brain uptake values of 7.23 ± 0.47%ID/g and 1.55 ± 0.01%ID/g, 5- and 120-min post tail-vein injection, respectively, thus providing a 5 min/120 min clearance a ratio of 4.66. For comparison, brain uptake ratios of Florbetapir (2 min/2 h) and Florbetaben (2 min/4 h) in normal mice are 4.07 (%ID/g (brain): 2 min: 7.33 ± 1.54; 2 h: 1.80 ± 0.07; 7.33/1.80; 4.07) [11] and 5.0 (%ID/g (brain): 2 min: 4.77; 4 h: 0.95; 4.77/0.95; 5.02) [37], respectively. Therefore, the brain uptake clearance ratio of ^18^F-**7B** (5 min/120 min) is slightly superior to Florbetapir and comparable to that of Florbetaben in healthy mice. For an agent to be able to serve as an Aβ-imaging agent, literature precedents indicate that brain uptake ratio (%ID/g; the earliest time point; 2 to 5 min to that of the latest time point; typically for carbon-11; 30 to 60 min; and F-18, 2 h) of 3.5 or above could be considered as a benchmark for the ability of a given agent to cross the blood-brain barrier [38]. Additionally, brain uptake of a given imaging agent is a net function of several components, such as cerebral regional blood flow, BBB permeability, plasma radiotracer concentration, and free fractions of the radiotracer in plasma and in the brain. Additionally, the lipophilicity of a given compound reflects critical physicochemical trait for neuroimaging radiotracers due to its direct relationship to membrane permeability, solubility in water, and entropic contribution to binding. Literature precedents indicate that lipophilic drugs readily cross the BBB, although other chemical characteristics, including the number of hydrogen bonds, molecular weight, polar surface area, and molecular size, are also known to be critical to passive transport. Lipophilicity measured via LogP_OCT_, the partition coefficient for nonionized molecules between octanol and water, is a good indicator of a molecule to permeate the brain, and molecules possessing Log *P* values of 0.9 and 3.0 have been shown to cross the BBB [39]. Conversely, radiotracers that are too lipophilic can also bind plasma proteins and undergo fast metabolism and also display high nonspecific binding. ^18^F-**7B** demonstrates a log *P* value of 1.3 which is identical to that of PIB (1.3) but considerably lower than that of Florbetapir (2.4) and Florbetaben (3.22). The clearance ratio of 4.66 (%ID/g; 5 min/120 min; Table 1) provides further evidence for the ability of the radiotracer to traverse the BBB *in vivo* and is consistent with previous data using multiphoton imaging [23]. Additionally, the agent ^18^F-**7B** showed excretion from other critical organs over 2 h and slight defluorination as a function of time indicated by bone accumulation thus consistent with pharmacokinetic profiles of other FDA-approved agents [11, 15, 16].

**Table 1:** **Biodistribution data (%ID/g) of [** ^**18**^ **F]-7B in FVB mice (** ***n*** **= 3)**

**Organ (%ID/g)**	**5.00 min**	**30.00 min**	**60.00 min**	**120.00 min**
Blood	3.18 ± 0.23	2.51 ± 0.09	2.40 ± 0.17	1.86 ± 0.05
Brain	7.23 ± 0.47	2.27 ± 0.13	2.00 ± 0.15	1.55 ± 0.01
Bone	1.61 ± 0.20	2.08 ± 0.13	3.41 ± 0.37	5.27 ± 0.34
Liver	16.22 ± 1.43	5.21 ± 0.08	3.44 ± 0.20	2.73 ± 0.16
Kidney	6.72 ± 0.44	2.83 ± 0.09	2.20 ± 0.07	1.59 ± 0.03

%ID, percentage injected dose.

**Figure 4:**
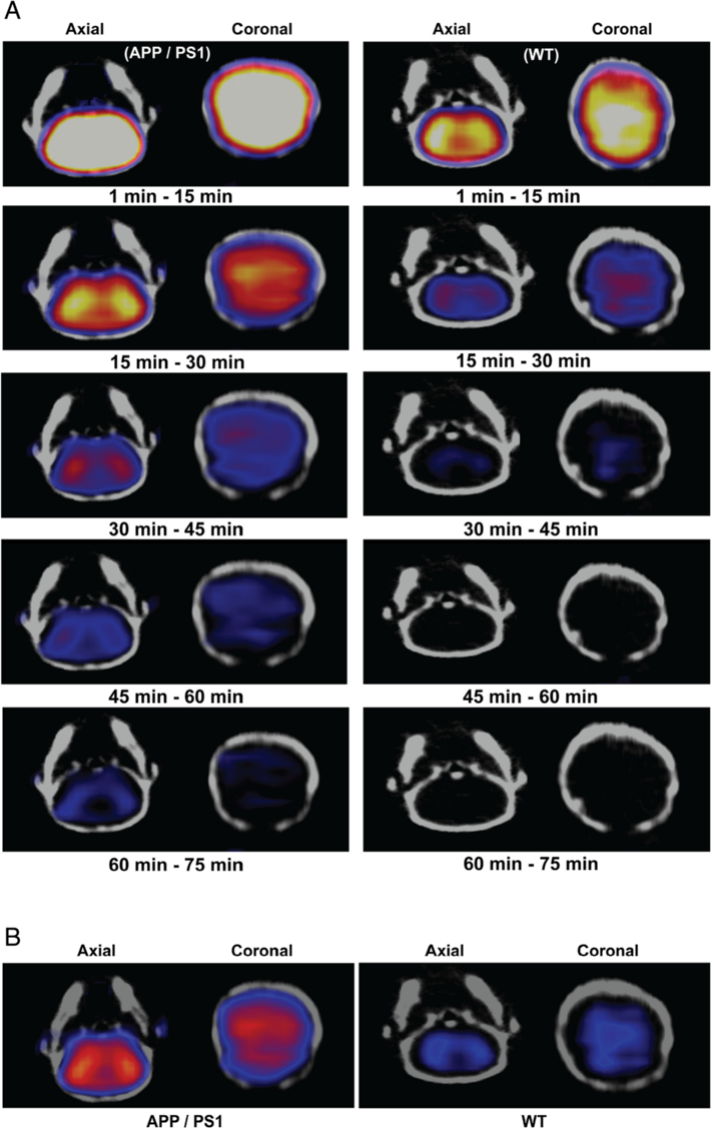
MicroPET/CT imaging. APP/PS1 and WT mice (22.6 months old; *n* = 3; closely age-matched) were injected intravenously with HPLC-purified [^18^F]-**7B** (12.95 MBq). Representative PET static images of brain (axial and coronal view) were obtained from 1- to 75-min post-intravenous injection and co-registered with CT for an anatomical reference. **(A)** Axial and coronal images of the transgenic and wild-type mice post-intravenous injection of the radiotracer. **(B)** Axial and coronal images of the transgenic and wild-type mice 15- to 45-min post-intravenous injection of the radiotracer Left: APP/PS1 mouse; Right: WT mouse. Note consistent higher retention of [^18^F]-**7B** in the brains of APP/PS1 (Left) compared with WT counterpart (Right). APP, amyloid precursor protein; PS1, presenilin-1; WT, wild type.

**Figure 5:**
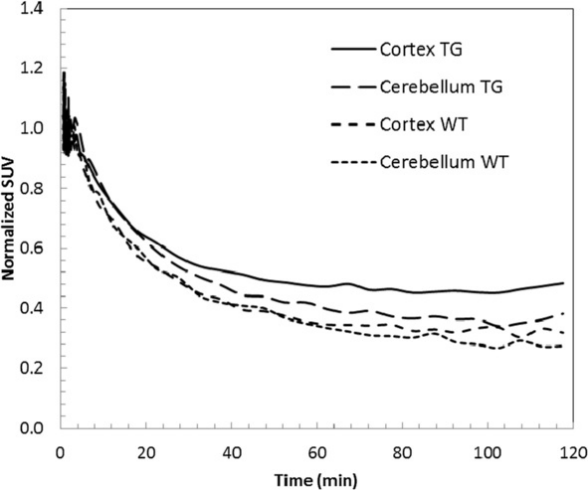
Time-activity curves (TACs) showing kinetics of [^18^F]-**7B** obtained from ROI of transgenic and WT mice. The SUV data were additionally normalized to the average activity measured in those regions between 1 and 2 min after injection. The normalized SUV data indicate a higher retention of the tracer in cortex of transgenic mice compared with its WT counterpart following a clearance of the unbound tracer, a pharmacokinetic profile consistent with binding of the agent ([^18^F]-**7B**) with Aβ plaques in transgenic mice. TG, ; WT, wild type; SUV, Standard Uptake Value.

Transgenic mice expressing mutated forms of the gene for the human amyloid precursor protein (hAPP) show a marked elevation in Aβ-protein levels and their deposition in the cerebral cortex and hippocampus [40], which represent neuropathological hallmarks similar to those observed in human AD brains. It is also noteworthy that presenilin-1 (PS1) mutant transgenic mice show increased Aβ_1-42_ peptide formation, thus augmenting amyloid deposition in Tg2576 APP mice at 6 months of age [41]. Therefore, the double-transgenic mice having co-expression of these mutated genes (APP^+/−^/PS1^+/−^) exhibit a strikingly accelerated accumulation of Aβ deposits compared with the single APP transgenic counterparts [42, 43]. Noticeably, several Aβ ligands [44–46] and disease-modifying therapeutics have been investigated for their efficacy using APP^+/−^/PS1^+/−^ transgenic models [47, 48]. Previously, we have shown via multiphoton imaging the pharmacokinetics of **7A** in brains of transgenic mice at the highest resolution and its ability to penetrate the BBB to label Aβ plaques in brain parenchyma and blood vessels (CAA) [23]. Additionally, the low level of background fluorescence from residual retention of **7A** in nearby brain regions suggested high signal-to-background ratios thus desirable of PET imaging *in vivo*. While fluorescence imaging offers high-resolution spatial localization of an imaging probe within a narrow field of view, the PET imaging provides its assessment at a relatively lower resolution within the whole organ. Therefore, it is necessary to interrogate sensitivity of the radiotracer for detecting Aβ *in vivo* at concentrations relevant for clinical nuclear imaging. To directly access the potential of ^18^F-**7B** to bind Aβ plaques *in vivo*, we performed microPET/CT imaging in age-matched APP^+/−^/PS1^+/−^ mice (*n* = 3) compared to their WT counterparts, 5-min to 2-h post tail-vein injection. For any given agent to serve as an Aβ-targeted agent, a pharmacokinetic model would involve an initial high and equal influx of the tracer into the brains of transgenic and WT mice, followed by clearance of the radiotracer from brains of WT mice, thus demonstrating differential retention in regions of brains, as the agent binds to Aβ plaques in transgenic mice. Given that initial brain influx would be expected to be the same in both mouse models, and differential retention of ^18^F-**7B** in transgenic mice could result from its binding to Aβ plaques and clearance of the unbound tracer as a function of time, we therefore used dynamic PET imaging over 1 to 75 min to interrogate regional localization of ^18^F-**7B** within the brains of transgenic and WT mice. Preliminary microPET/CT imaging indicates a higher retention of ^18^F-**7B** in the frontal cortex of transgenic mice compared with their WT age-matched counterparts (Figure 4A,B) indicating its ability to traverse the BBB and label Aβ plaques in transgenic mice**.** Similar to ^11^C-PIB [46, 49] and Florbetaben [45], ^18^F-**7B** shows also considerable retention in extracerebral regions, such as nasal and eye cavities. Furthermore, coupled with binding affinity and pharmacokinetic profiles of a given imaging agent, the presentation of different forms (diffuse and compact forms) of Aβ and the degree of fibrillary plaques can also impact signal-to-noise ratios (SNRs), thus influencing image quality. Additionally, Florbetaben, PiB, and Amyvid are known to preferentially bind fibrillary plaques, while **7A** shows binding to both diffuse and fibrillary plaques, though with a lower affinity compared with approved tracers. Given these variations in targeting profiles and binding affinity, slightly lower SNR was observed using microPET imaging. For evaluation of kinetics, time-activity curves were obtained in the cortex and cerebellum (reference) regions of transgenic and WT mice (Figure 5). The data show a consistently higher retention of ^18^F-**7B** (40 min post-injection) in the cortex of transgenic mice compared to their age-matched WT controls (Figure 5), and these observations are in accord with other FDA-approved agents in rodents [44–46]. For further evaluation of imaging potential of ^18^F-**7B** and analysis of SNRs *in vivo*, microPET imaging of transgenic mice as a function of aging profiles is under investigation. Overall, these PET data show excellent correlation with earlier studies employing its unlabeled counterpart **7A** via multiphoton imaging [23].

## Conclusions

In summary, a heterocyclic PET radiopharmaceutical ^18^F-**7B** was synthesized and analytically characterized. The radiotracer demonstrates concentration dependent saturable binding to both Aβ_1-42_ fibrils and AD homogenates, while also indicating 3.4-fold higher binding affinity for AD homogenates compared with Aβ_1-42_ fibrils. The binding affinity of ^18^F-**7B** with AD homogenates is slightly inferior to that of Flutemetamol and Florbetapir and comparable to Florbetaben. ^18^F-**7B** demonstrated labeling of Aβ plaques in cortex of AD brain sections and the binding to Aβ was inhibited in the presence of its unlabeled counterpart (**7A**) indicating specificity of the probe. Pharmacokinetics data in normal mice indicate a higher initial influx into the brain, followed by clearance due to a lack of targeted plaques within the brains of normal mice, an important factor that could determine critical target/background ratios. Finally, pilot microPET/CT imaging show a sustained higher retention of ^18^F-**7B** in brains of transgenic mice compared with their WT counterpart, consistent with the binding of ^18^F-**7B** to Aβ plaques in transgenic mice thereby supporting multiphoton-imaging data (using **7A**) reported earlier [23]. These data provide a platform scaffold for further optimization to develop PET tracers with enhanced affinity and specificity for noninvasive assessment of Aβ plaques and studies of Aβ pathophysiology *in vivo*.
